# Gonadotropin-mediated chemoresistance: Delineation of molecular pathways and targets

**DOI:** 10.1186/s12885-015-1938-x

**Published:** 2015-11-25

**Authors:** Suchismita Sahoo, Poonam Singh, Beneeta Kalha, Om Singh, Rahul Pal

**Affiliations:** Immunoendocrinology Lab, National Institute of Immunology, Aruna Asaf Ali Marg, JNU Complex, New Delhi, Delhi-110067 India

**Keywords:** Human chorionic gonadotropin, Ectopic secretion, Prognosis, Chemoresistance, Immunotherapy

## Abstract

**Background:**

Human chorionic gonadotropin (hCG) has essential roles in pregnancy. Reports linking hCG in non-trophoblastic tumors with poor patient prognosis has spurred interest in patho-physiological roles the hormone might play.

**Methods:**

The ability of hCG to prevent tumor cell death and sustain viability in the presence of chemotherapeutic drugs was assessed and potential synergies with TLR ligands explored. hCG-induced up-modulation of genes involved in chemoresistance was documented and targets validated by siRNA knock-down. Whether hCG could drive collaboration between tumor cells and macrophages in the production of IL-6 and consequent chemoresistance was assessed. The effects of concurrent anti-hCG immunization and chemotherapy on the growth of syngeneic murine tumors were evaluated.

**Results:**

hCG maintained basal levels of cytokine secretion by tumor cells exposed to chemotherapeutic drugs, and enhanced viability and proliferation; pre-treatment with hCG also decreased apoptosis, as assessed by Annexin-V binding and the cleavage of caspase 3. While co-incubation with hCG along with several TLR ligands mediated heightened chemo-resistance, TLR-2/6 and TLR-9 ligands increased the phosphorylation of JNK, and TLR-2 and TLR-8 ligands the phosphorylation of ERK in presence of hCG and curcumin, providing evidence of tri-molecular synergy. The hormone increased the transcription and/or expression of molecular intermediates (SURVIVIN, HIF-1α, PARP-1, Bcl-2, c-FLIP, KLK-10, XIAP, c-IAP-1) associated with chemo-resistance and increased levels of stress modulators (PON2, HO-1, HSP27 and NRF-2). siRNAs to SURVIVIN, NRF-2, HO-1 and HIF-1α attenuated hCG-mediated chemo-resistance. hCG-conditioned tumor cell supernatants induced heightened secretion of IL-6 and TNF-α from peripheral blood adherent cells and secreted IL-6 imparted chemo-resistance to naïve tumor cells. Co-administration of curcumin along with an anti-hCG vaccine (hCGβ conjugated to Tetanus Toxoid (TT)) to mice carrying syngeneic tumors resulted in significantly enhanced benefits on animal survival; synergy was demonstrated between anti-hCG antibodies and curcumin in the reduction of tumor cell viability.

**Conclusions:**

The data suggest that hCG, via direct as well as collaborative effects with TLR ligands and accessory cell-secreted cytokines, mediates chemo-resistance in gonadotropin-sensitive tumors and outlines the potential benefits of combination therapy.

**Electronic supplementary material:**

The online version of this article (doi:10.1186/s12885-015-1938-x) contains supplementary material, which is available to authorized users.

## Background

Chorionic gonadotropin, a glycoprotein hormone specific to primates, is possibly the first maternal indicator of pregnancy. It extends the life of the corpus luteum to elicit the continued secretion of progesterone [1], enables implantation and trophoblast invasion, and supports angiogenesis [2, 3].

Several non-trophoblastic tumors have been shown to express human chorionic gonadotropin (hCG) or its constituent subunits [4–6]. Data are suggestive of hCG playing a role in tumor growth by autocrine/paracrine mechanisms. Incubation of bladder cancer cells with hCGβ leads to dose-dependent proliferation [7], and tumor-derived hCG is associated with unresponsiveness to treatment and decreased survival [8, 9]. Further, hCGβ-positive patients respond poorly to radiotherapy [10] and its presence is associated with an aggressive cancer phenotype and high metastatic potential [11]. Evidence for a pro-tumorigenic role for hCG has also been provided by transgenic models; mice expressing hCG develop gonadal neoplasia and extra-gonadal tumors [12].

Chemo-resistance can result from numerous mechanisms, including decreased intracellular drug concentrations, an augmented ability to repair DNA damage or endure stress, as well as the prevention of cell death. TLR ligands [13], HIF-1α [14], Inhibitor of Apoptotic Proteins [15] and pro-inflammatory cytokines [16] have been linked with chemo-resistance and tumorigenesis. The tumor microenvironment, including associated non-transformed cells, can also contribute towards creation of a pro-tumorigenic milieu [17].

Results obtained during this study support the notion that exogenous gonadotropin can enhance tumor cell growth and viability while additionally mediating chemo-protection, and also identify potential molecular intermediates. Further, the data also suggests that third-party influences, such as enhanced signaling via toll-like receptors and inflammatory cytokines secreted by non-transformed accessory cells, can collaborate to enhance hCG-mediated effects. Finally, evidence is provided to suggest that potent anti-hCG vaccination, as an adjunct to conventional chemotherapy, may hold promise in the treatment of cancers rendered chemo-resistant due to the action of gonadotropin.

## Methods

### Ethics statement

This study was carried out in strict accordance with protocols approved by the Institutional Animal Ethics Committee (IAEC) of the National Institute of Immunology (IAEC Number: 231/10) and according to national and international ethical guidelines.

### Animals

Six week-old inbred female C57BL/6 mice were obtained from the Small Animal Facility of the National Institute of Immunology, New Delhi.

### hCG, drugs, cell lines

Though recombinant hCG has been established to be biologically potent in conventional assays (on reproductive tissues and processes) by several investigators, it contains carbohydrates that are distinct from those on native hCG due to variations in the extent of oligosaccharide branching and sialylation. Since this study aimed to evaluate the chemo-protective effects of hCG on tumor cells (which have thus far not been described in any detail), it was reasoned appropriate to employ native hCG so as to expose cells to as natural a biological stimulus as possible. Native hCG (Wyong Biologicals) was subjected to physical (by SDS-PAGE and HPLC), immunological (by a hCGβ-specific radioimmunossay and Western blot) and biological (by radioreceptor assay and Leydig cell bioassay) characterization; the preparation did not contain significant amounts of free hCGβ and was independently assessed to express an activity of ≅ 11,000–13,000 IU/mg against relevant hCG standards, the same range as reported for recombinant hCG when the contribution of carbohydrates is taken into account. hCG was employed over a range of concentrations (10 μg/ml–100 ng/ml or ≅ 110 IU/ml–1.1 IU/ml).

Curcumin was obtained from Sanat Products; Tamoxifen, Etoposide and 5-Fluorouracil (5-FU) were obtained from Enzo Life Sciences. Human (Colorectal: COLO-205 (CCL-222); Lung: ChaGo-K-1 (HTB-168)) and murine (Lewis Lung: LLC1 (CRL-1642); Melanoma: B16 (CRL-6323); Myeloma: SP2/O (CRL-1581)) cell lines were obtained from ATCC and used within six months of receipt or resuscitation. Human cell lines were authenticated by STR at ATCC as per convention. All cell lines were also authenticated by assessment of phenotypic, morphological and growth characteristics, and were mycoplasma-free. Cells were cultured in RPMI 1640 (Life Technologies) or DMEM along with 10 % fetal bovine serum (Biological Industries).

### Effects of hCG on tumor cell viability, proliferation

B16, COLO-205, SP2/O and ChaGo-K-1 cells (5 × 10^4^) were incubated with hCG for 24 h. 50 μl of 3-(4, 5-Dimethylthiazol-2-yl)-2, 5-diphenyltetrazolium bromide (MTT, Sigma) was then added and the reaction arrested using DMSO. Absorbance was assessed at 550 nm. Data (for this and subsequent MTT experiments) was plotted as percentage viability compared with relevant controls. Tumor cells pre-exposed to hCG for 24 h were incubated with [^3^H]-Thymidine (0.5 μCi/well), followed by incubation for an additional 18 h. Cell-associated radioactivity was assessed in a β-counter.

### Effects of hCG on chemotherapeutic drug-induced loss of tumor cell viability, apoptosis

COLO-205, ChaGo-K-1, SP2/O and LLC1 cells pre-exposed to hCG for 24 h were treated with curcumin (10 μM - 160 μM, Sigma) or tamoxifen (0.1 μM - 40 μM, Sigma) for 24 h and 48 h respectively, and MTT assays were carried out. In parallel experiments, reactivity of cells to Annexin-V was determined by flow cytometry; briefly, cells were incubated with Annexin-V-FITC (BD Biosciences) for 15 m and run on a flow cytometer (BD-LSR; BD Biosciences). Data was analysed using WinMDi 2.9 software.

### Effect of hCG on activation of caspase-3

ChaGo-K-1 cells were incubated with hCG for 24 h and subsequently with curcumin (40 μM) for 24 h. Conversion of pro-caspase 3 to active (cleaved) caspase 3 was assessed using an apoptosis array kit (R&D Systems). Reactive spots were visualized by enhanced chemiluminescence (ECL; Biological Industries).

### Combined effects of hCG and TLR ligands on chemotherapeutic drug-induced loss of tumor cell viability and on cell signaling

#### Cell Viability

COLO-205 cells pre-exposed to hCG for 24 h were incubated with individual TLR ligands (Invivogen; TLR 1/2: Pam3CSK4 (1 μg/ml); TLR2: HKLM (10^8^ cells/ml), TLR3: Poly I:C (10 μg/ml); TLR4: LPS (10 μg/ml); TLR5: Flagellin (10 μg/ml); TLR2/6: FSL-1 (1 μg/ml), TLR7: Imiquimod (5 μg/ml); TLR8: ssRNA40 (10 μg/ml); TLR9: ODN2006 (5 μM)) for 24 h. Cellular viability upon subsequent treatment with curcumin (at 40 μM) was assessed at 24 h.

#### Cell Signaling

COLO-205 cells were incubated with hCG for 24 h prior to incubation with individual TLR ligands (at the concentrations indicated above) for 24 h. Curcumin (at 40 μM) was then added and a further incubation carried out for 24 h. Cells were then harvested and lysates probed with monoclonal antibodies specific for total and phosphorylated ERK and JNK (Santacruz Biotech) by Western blot. Reactive moieties were visualized by enhanced chemiluminescence (ECL; Biological Industries).

### Mediators of hCG-induced chemo-resistance

#### Identification

1 μg total RNA, obtained from ChaGo-K-1 cells pre-exposed to hCG, was reverse transcribed into cDNA using oligo-dT and reverse transcriptase (Promega). Primers for amplification of human *PARP-1, BCL-2, HIF-1α, SURVIVIN, KLK-10, c-FLIP*, different *TLRs* and β-ACTIN (as control) are listed in Additional file 1: Figure S1. For PCR, a 15 m denaturation step at 95 °C was followed by 35 cycles of three steps each: 95 °C for 1 m, annealing for 1 m, extension at 72 °C for 1 m, followed by final extension at 72 °C for 10 m.

Cellular lysates, obtained from ChaGo-K-1 cells pre-exposed to hCG, were electrophoresed and subsequently transferred onto nitrocellulose membranes (mdi), and probed with monoclonal antibodies specific to *PARP-1, BCL-2, HIF-1α, SURVIVIN, KLK-10* and *c-FLIP*. (Santacruz Biotech); reactivity, subsequent to the addition of secondary antibodies (Jackson ImmunoResearch), was visualized by enhanced enzymatic chemi-luminescence. Expression levels of the molecules subsequent to the incubation of ChaGo-K-1 cells with hCG were also assessed by flow cytometry; cells were permeabilized before incubation with specific antibodies for 1 h at 4 °C. After addition of FITC-conjugated secondary antibodies (Jackson ImmunoResearch), samples were run on a flow cytometer and data analyzed by WinMDi 2.9 software.

ChaGo-K-1 cells were incubated with hCG for 24 h and subsequently to curcumin (40 μM) for 24 h. Effects on thirty five mediators involved in apoptosis and stress regulatory responses were assessed using an apoptosis array kit (R&D Systems). Reactive spots were visualized by enhanced chemiluminescence.

#### Validation

ChaGo-K-1 cells were transiently transfected with either control scrambled RNA (scRNA) or siRNA directed against *HIF-1Α, SURVIVIN, NRF-2* and *HO-1* (Santacruz Biotech). Briefly, scRNA or siRNA was diluted in transfection medium to 30 pM, 60 pM or 120 pM. The solution was mixed with transfection reagent, incubated for 30 m at room temperature and overlaid on ChaGo-K-1 cells, following which an incubation was carried out for 6 h at 37 °C. Medium supplemented with 20 % FCS was added and a further incubation carried out for 16 h. Cells harvested from two parallel experiments were assayed for decrease in mRNA (by semi-quantitative RT-PCR) and protein (by Western blot) expression. The ability of hCG to mediate chemo-resistance in transfected cells was then assessed in a cell viability assay as outlined above.

### Assessment of the role of IL-6 in hCG-induced chemo-resistance

ChaGo-K-1 and COLO-205 cells were incubated with recombinant IL-6 (at 50 ng/ml; R&D Systems) for 6 h and subsequently incubated with curcumin (40 μM) for 24 h. Viability was assessed by MTT.

hCG tumor-conditioned medium (obtained upon incubation of ChaGo-K-1 and COLO-205 with hCG for 24 h) was incubated with peripheral blood adherent cells (PBACs; obtained upon plastic adherence of human PBMCs) for 24 h. Levels of IL-6 and TNF-α in PBAC supernatants were determined by ELISA (eBiosciences). The ability of such PBAC supernatants to mediate resistance to curcumin (at 40 μM) in naïve ChaGo-K-1 and COLO-205 cells was assessed by MTT; the contribution of elicited IL-6 to these effects was assessed using anti-IL6 neutralizing antibodies (500 ng/ml; R&D Systems).

### Effects of anti-hCG immunization and chemotherapy in tumor-bearing mice

#### Vaccine formulation

hCGβ was conjugated to tetanus toxoid (TT) in a molar ratio of 6:1 using the cross-linker sulfosuccinimidyl 6-[3^׳^ (2-pyridyldithio)-propionamido] hexanoate (LC-sulpho-SPDP; Pierce) as previously described [18]. The hCGβ content in the conjugate was estimated by radioimmunoassay. Briefly, increasing amounts (0.125 ng to 4 ng) of hCG or dilutions of the hCGβ-TT conjugate were incubated at 4 °C for 18 h with a murine anti-hCGβ specific monoclonal antibody in the presence of ^125^I-hCG (≅15,000 dpm; specific activity: 40–60 μCi/μg) and 4 % normal horse serum. The antibody bound fraction was precipitated by adding PEG 8000 (12.5 % final concentration), separated by centrifugation at 1500 g at 4 °C for 20 m and counted for radioactivity. The concentration of hCGβ in the conjugate was estimated with reference to the standard curve.

hCGβ-TT was adsorbed on Alhydrogel (Superfos; 1 mg protein/ml slurry) by incubation on an end-to-end rocker at 4 °C for 16 h. Adsorption efficiency was greater than 95 %.

*Mycobacterium indicus pranii* (MIP) was grown in Middlebrook 7H9 media (BD Difco) supplemented with 10 % albumin-dextrose complex enrichment (BD Difco), 0.02 % glycerol, and 0.05 % Tween-80. Bacteria were killed by autoclaving at 121 °C at a pressure of 15 lb/in^2^ for 20 m.

#### Intervention

Groups of female inbred C57BL/6 mice were subcutaneously implanted with syngeneic LLC1 cells (4 × 10^4^ cells/mouse). On the same day, mice received a subcutaneous injection of 2 μg alhydrogel-adsorbed hCGβ-TT emulsified in Incomplete Freund’s Adjuvant (IFA; Life Technologies) along with MIP (10^8^ cells; intramuscular), hereafter referred to as immunotherapy. Three injections were administered at monthly intervals. A second group of animals was co-administered curcumin (2.5 mg/kg; intra-peritoneal, thrice weekly) along with immunotherapy. Additional groups of animals were treated with curcumin, the vehicles, or were left untreated. Tumor volumes (4/3πr^3^; r = (length + width)/4) were recorded at periodic intervals.

### Assessment of synergy between anti-hCG antibodies and curcumin

*In vitro* experiments were conducted to demonstrate whether anti-hCG antibodies and curcumin exerted synergistic effects on ChaGo-K-1 cells. Anti-hCG antibodies (0–10 μg/ml) and curcumin (0–80 μM) were added alone and in combination to ChaGo-K-1 cells and an incubation carried out for 24 h. MTT assays were carried out as described above. Whether the effects obtained upon the combination of both moieties constituted synergy was calculated on the basis of the Combination Index (C.I.) as:

$$ \frac{{\mathrm{C}}_{50}\mathrm{antibody}}{{\mathrm{IC}}_{50}\mathrm{antibody}}+\frac{{\mathrm{C}}_{50}\mathrm{curcumin}}{{\mathrm{IC}}_{50}\mathrm{curcumin}} $$

where IC_50_ antibody and IC_50_ curcumin refer to respective concentrations resulting in 50 % inhibition in MTT assays (when added alone), and C_50_ antibody and C_50_ curcumin refer to respective antibody and curcumin concentrations (when added in combination) resulting in 50 % inhibition in MTT assays. Values of C.I. less than one signify synergy, equal to one additivity, and greater than one, antagonism [19].

### Statistical Analysis

Data are expressed as arithmetic mean ± SD. Statistical significance was assessed using the unpaired Student’s *t*-test; the data distribution in the samples assayed was normal. Animal survival was analyzed by the method of Kaplan-Meier.

## Results

### Effects of hCG on tumor cell viability, proliferation

**Fig. 1:**
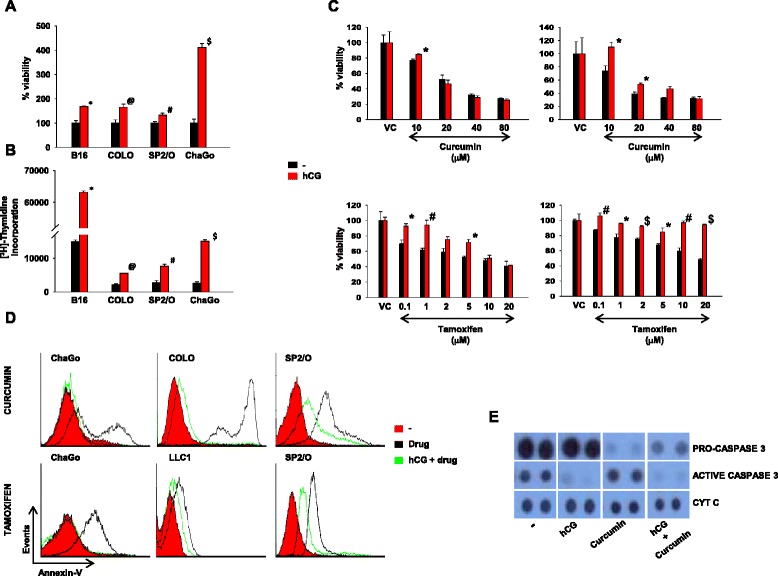
hCG prevents chemotherapeutic drug-induced decrease in tumor cell viability and apoptosis. **a** Cell viability analysis on B16, COLO-205, SP2/O and ChaGo-K-1 cells 24 h after hCG (10 μg/ml or ≅ 110 IU/ml) exposure. Means ± SEM of four independent experiments (with three replicates) are shown. For each cell line, data has been individually normalized to cells plus medium. (*p < 0.003, ^@^p < 0.03, ^#^p < 0.04, ^$^p < 0.0003 vs cells plus medium). **b** Proliferative responses of B16, COLO-205, SP2/O and ChaGo-K-1 cells 24 h after hCG exposure. Means ± SEM of three independent experiments (with three replicates) are shown (*p < 0.0001, ^@^p < 0.0001, ^#^p < 0.0001, ^$^p < 0.0001 vs cells without hCG). **c** Cell viability analysis of COLO-205 (left top and bottom panels) and SP2/O (right top and bottom panels) cells after pre-incubation with either medium or hCG for 24 h and then exposed to different concentrations of curcumin (for 24 h) or tamoxifen (for 48 h). VC: Vehicle control. Means ± SEM of four independent experiments (with three replicates) are shown. For each cell line and for both control cultures and cultures containing hCG, data has been individually normalized to cells plus vehicle control (VC). (*p < 0.04, ^#^p < 0.009, ^$^p < 0.0009 vs cells not exposed to hCG at the same drug concentration). **d** Reactivity of ChaGo-K-1, COLO-205, SP2/O and LLC1 cells towards Annexin-V-PE by flow cytometry post-exposure to two chemotherapeutic drugs (black profiles). In test cultures (green profiles), cells were exposed to hCG before addition of the drugs. Red profiles indicate secondary antibody controls. Representative profiles of three independent experiments are shown. **e** Levels of pro-caspase 3 and caspase 3 in ChaGo-K-1 cells treated with hCG, curcumin or with hCG + curcumin. Cytochrome c (CYT C) was employed as loading control

B16, COLO-205, SP2/O and ChaGo-K-1 cells were incubated with hCG for 24 h; viability and proliferative responses were subsequently assessed. Human and murine tumor cells exposed to hCG demonstrated increased viability as well as increased rates of cell proliferation (Fig. 1a and b).

### Effects of hCG on chemotherapeutic drug-induced loss of tumor cell viability

Based on the association of hCG with increased chemo-resistance [9,20], whether hCG can directly induce chemo-resistance in tumor cells was ascertained. hCG pre-exposure resulted in a significant reduction in drug-induced decrease in viability in both human and murine tumor cells, though individual responses varied (Fig. 1c); while hCG protected COLO-205 cells from only low to moderate concentrations of curcumin and tamoxifen, SP2/O cells exposed to even high concentrations of tamoxifen remained significantly protected.

### Effects of hCG on chemotherapeutic drug-induced apoptosis of tumor cells

Drug resistance is a multifactorial process and is characterized by dysregulation of the equilibrium between cell survival and apoptosis [21]. Whether hCG can effect chemotherapeutic drug-induced apoptosis of tumor cells was assessed; four tumor cell lines (COLO-205, SP2/O, ChaGo-K-1, LLC1) and two drugs (curcumin, tamoxifen) were employed. Pre-exposure to hCG dramatically reduced the binding of Annexin-V in cells incubated with the drugs (Fig. 1d).

Incubation of ChaGo-K-1 cells with curcumin significantly reduced levels of pro-caspase 3 compared to control cells, as expected. Significantly, pre-incubation with hCG reduced levels of active (cleaved) caspase 3. In cells incubated with both curcumin and hCG, levels of pro-caspase 3 were higher and levels of active (cleaved) caspase 3 lower compared to cells treated with curcumin alone (Fig. 1e). These results suggest that hCG mediates significant anti-apoptotic effects.

### Effects of hCG on TLR expression and the combined effects of hCG and TLR ligands on chemo-resistance

**Fig. 2:**
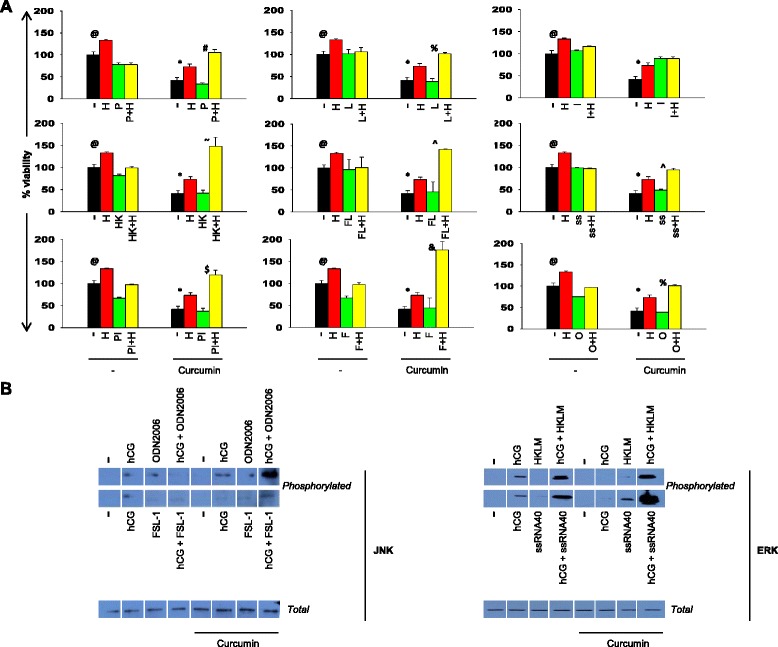
hCG and TLR ligands synergistically enhance chemo-resistance. **a** Cell viability analysis on COLO-205 cells incubated with hCG (for 24 h), individual TLR ligands (for 24 h) and curcumin (for 24 h) alone and in combination. H: hCG; P: Pam3CSK4; HK: HKLM; PI: Poly I:C; L: LPS; FL: Flagellin; F: FSL-1; I: Imiquimod; ss: ssRNA40; O: ODN2006. Means ± SEM of three independent experiments (with three replicates) are shown. In each case, data has been individually normalized to control cultures. (^@^p < 0.01 vs control cells, *p < 0.03 vs cells + curcumin, ^#^p < 0.04, ^%^p < 0.02, ^p < 0.0005, ^&^p < 0.008 vs hCG + curcumin). **b** Western blot analysis of the phosphorylation of JNK (left panel) and ERK (right panel) in COLO-205 cells exposed hCG, individual TLR ligands and curcumin, individually or in combination, as indicated, in the manner described above

Increasing evidence points to a potential role for TLRs in tumorigenesis and chemo-resistance [13, 22]. The combined effects of hCG and TLR ligands on chemo-resistance was assessed. Incubation with hCG led to increases in mRNA and protein levels of TLR1, TLR3, TLR8 and TLR9 in COLO-205 cells (Additional file 2: Figure S2). Treatment of these cells with TLR ligands (in the presence or absence of drugs) did not affect viability over respective control cultures. COLO-205 cells pre-exposed to hCG, then to individual TLR ligands and subsequently to curcumin, demonstrated increased viability in comparison to appropriate controls; interestingly, in several instances, the addition of TLR ligands along with hCG resulted in greatly enhanced chemo-protection (Fig. 2a). Qualitatively similar effects were observed when tamoxifen was employed as the chemo-therapeutic agent (Additional file 3: Figure S3).

To gain further insight into the observed enhancement in chemo-protective effects, the phosphorylation of ERK and JNK upon the addition of hCG, individual TLR ligands and curcumin (alone and in combination) was assessed; prominent data is discussed here. While individual addition of hCG and the TLR-9 ligand ODN2006 induced JNK phophorylation, these moieties in combination and in the additional presence of curcumin induced a synergistic increase. Similar synergism was observed when the TLR6 ligand FSL-1 was added along with hCG and curcumin. While hCG induced the phosphorylation of ERK and ligands for both TLR-2 and TLR-8 (HKLM and ssRNA40 respectively) were ineffective (at 48 h post stimulation), hCG acted in synergy with both these ligands to enhance ERK phoshorylation. The addition of curcumin to hCG and ssRNA40 resulted in further enhancement (Fig. 2b). To summarize, both bimolecular (involving some TLR ligands and hCG) as well as tri-molecular (involving some TLR ligands, hCG and curcumin) synergies were observed, the potential significance of which is discussed below.

### Identification and validation of potential mediators of hCG-induced chemo-resistance

**Fig. 3:**
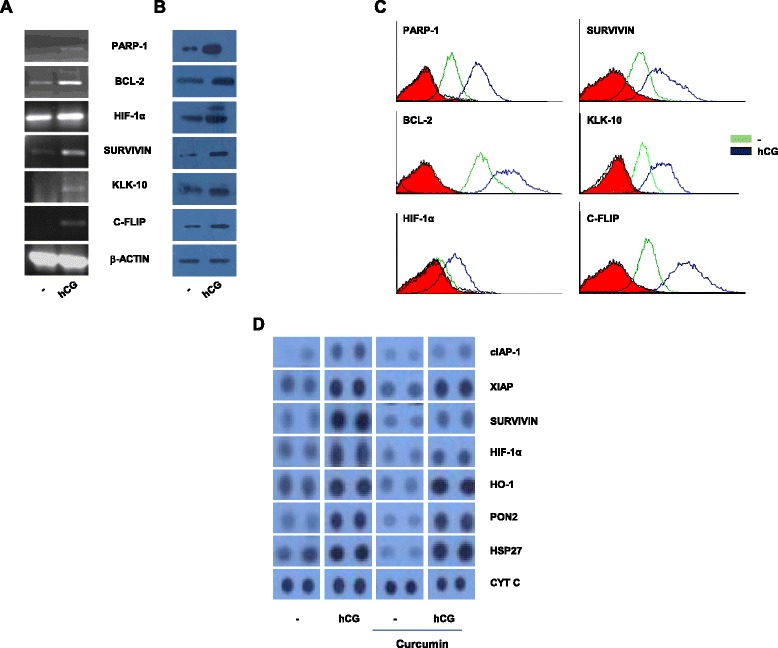
hCG increases mRNA and expression levels of molecules associated with chemo-resistance, apoptosis and cell stress in tumor cells. **a** Semi-quantitative RT-PCR of molecules associated with chemo-resistance upon incubation of ChaGo-K-1 cells with medium or medium supplemented with hCG. β-ACTIN was employed as loading control. **b** Western blot of molecules associated with chemo-resistance upon incubation of ChaGo-K-1 cells with medium or medium supplemented with hCG. β-ACTIN was employed as loading control. **c** Flow cytometric analysis of molecules associated with chemo-resistance upon incubation of ChaGo-K-1 cells with medium or medium supplemented with hCG. Cells were permeabilized and stained with the respective antibodies. Filled red profiles indicate secondary antibody controls, green profiles indicate control cells and blue profile cells incubated with hCG. Data in A-C representative of four independent experiments. **d** Array dot blot analysis on ChaGo-K-1 cells incubated with medium, hCG, curcumin, or with hCG + curcumin. Expression levels of proteins associated with inhibition of apoptosis, chemo-resistance and protection from cell stress are shown. Cytochrome c (CYT C) was employed as loading control

Message and expression levels of molecules known to be associated with chemo-resistance and also known to be modulated by hCG (for the most part, in reproductive tissues) were evaluated. mRNA and protein levels of PARP-1, BCL-2, HIF-1α, SURVIVIN, KLK-10 and c-FLIP increased in ChaGo-K-1 cells upon hCG treatment (Fig. 3a-c). Several of these factors were transcriptionally and translationally upregulated in COLO-205 cells upon hCG treatment as well (Additional file 4: Figure S4). A dot blot array capable of detecting thirty five apoptosis- and stress regulatory response-related proteins was employed to confirm and extend these findings. ChaGo-K-1 cells incubated with hCG demonstrated increases in the levels of cIAP-1, XIAP and SURVIVIN (inhibitors of apoptotic proteins), HIF-1α, HO-1 (associated with chemo-resistance and tumorigenesis), and PON2 and HSP27 (associated with protection from cell stress). Incubation with curcumin alone caused observable decreases in the levels of several of these molecules. Significantly, levels of these chemo-protective molecules remained high even in cells treated with apoptosis-inducing doses of curcumin in the presence of hCG (Fig. 3d).

**Fig. 4:**
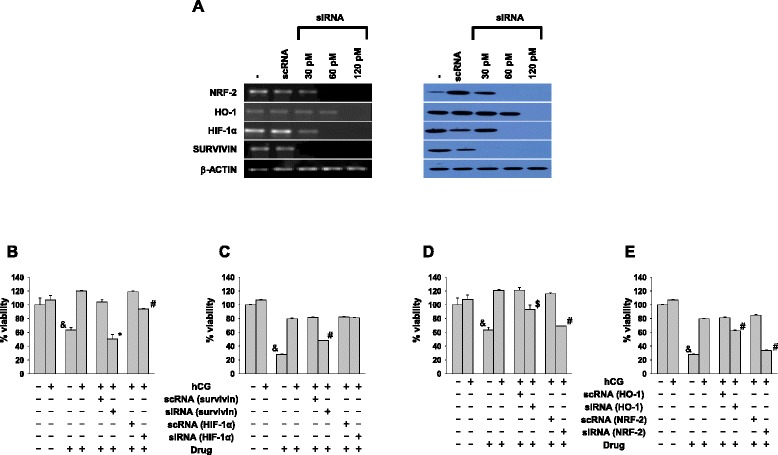
Down-modulation of SURVIVIN, HIF-1α, HO-1 and NRF-2 decreases hCG-induced chemo-resistance. **a** mRNA levels by semi-quantitative RT-PCR (left panel) and protein levels by Western blot (right panel) of SURVIVIN, HIF 1-α, HO-1 and NRF-2 upon transfection of respective siRNAs in ChaGo-K-1 cells. scRNA: scrambled RNA control. β-ACTIN was employed as loading control. **b-e** Cell viability analysis of ChaGo-K-1 cells individually transfected with siRNAs against SURVIVIN, HIF 1-α, HO-1 and NRF-2 incubated with hCG and later exposed to (**b**, **d**) tamoxifen or (**c**, **e**) curcumin. scRNA: scrambled RNA control. For **b**-**e**, means ± SEM of four independent experiments (with three replicates) are shown. In each case, data has been individually normalized to control cultures. (^&^p < 0.0003 vs drug + hCG; *p < 0.003, ^#^p < 0.0003, ^$^p < 0.03 vs hCG + respective scRNA + respective drug)

HIF-1α, HO-1 and SURVIVIN were selected for further study, based on the weight of evidence linking them with chemo-resistance and tumorigenesis [23]; further, NRF-2 was also chosen, given its role up-stream of these molecules [24]. Specific siRNAs designed to target these molecules decreased transcript and protein levels in a dose-dependent manner (Fig. 4a). As anticipated, decreased expression of SURVIVIN and HO-1 levels were observed when siRNA against HIF-1α and NRF-2 was respectively employed (Additional file 5: Figure S5).

To investigate the roles of HIF-1α, HO-1, NRF-2 and SURVIVIN in hCG-induced chemo-resistance, ChaGo-K-1 cells transfected with individual siRNAs were incubated with hCG and later exposed to either tamoxifen or curcumin. Silencing the proximal factor NRF-2 and the distal factor SURVIVIN either fully or substantially reversed the protection rendered by hCG against both drugs. Addition of siRNA to HO-1 or HIF-1α, on the other hand, had more modest or minimal effects, depending on the nature of the toxic insult (Fig. 4 b-e).

### Role of IL-6 derived from non-transformed cells in hCG-induced chemo-resistance

**Fig. 5:**
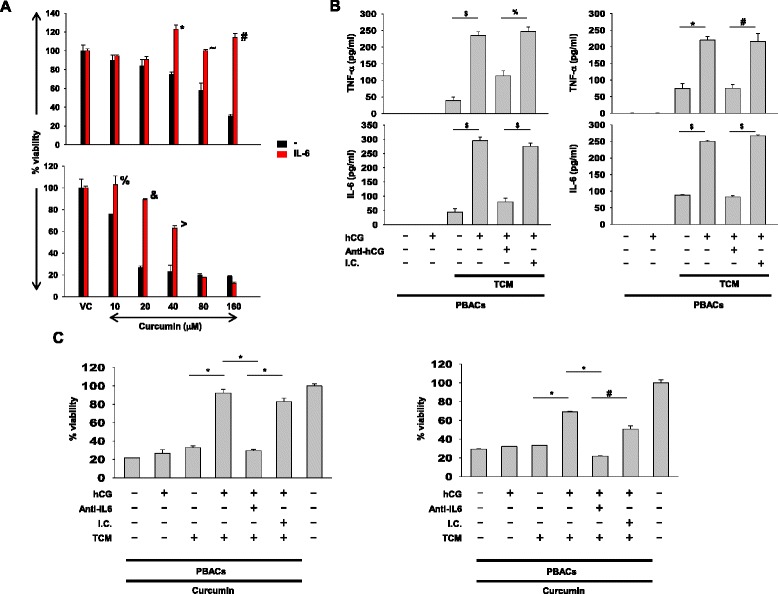
hCG-driven collaborative elicitation of IL-6 and consequent chemo-resistance. **a** Cell viability analysis on COLO-205 (top panel) and ChaGo-K-1 (bottom panel) cells pre-incubated with recombinant IL-6 and later exposed to curcumin. Means ± SEM of four independent experiments (with three replicates) are shown. For each cell line and for both control cultures and cultures containing hCG, data has been individually normalized to cells plus vehicle control (VC).(*p < 0.0009, ^~^p < 0.006, ^#^p < 0.0001, ^%^p < 0.03, ^&^p < 0.0001, ^>^p < 0.0004 vs cells not exposed to IL-6 at the same drug concentration). **b** Levels of TNF-α and IL-6 in supernatants of peripheral blood adherent cells (PBACs) exposed to hCG or to tumor-conditioned medium (TCM) derived from either control or from hCG-pre-incubated COLO-205 (left top and bottom panels) and ChaGo-K-1 (right top and bottom panels) cells. The effects of concurrent incubation with anti-hCG antibodies or isotype control (I.C.) antibodies are also shown (^$^p < 0.0001,*p < 0.0002, ^%^p < 0.0004, ^#^p < 0.0008). **c** Assessment of the ability of IL-6 (in supernatants of PBACs obtained upon incubation with hCG tumor-conditioned medium) to mediate resistance to curcumin in COLO-205 (left panel) and ChaGo-K-1 (right panel) cells. The relative efficacies of anti-IL-6 antibodies and isotype control (I.C.) antibodies to neutralize the protective effects of IL-6 are also shown. The right-most bars in each graph represent controls cultures in which only medium was dispensed to which data has been individually normalized. (*p < 0.0003, ^#^p < 0.001). For **b** and **c**, means ± SEM of three independent experiments (with three replicates) are shown

Previous literature documents the production IL-6 (a cytokine associated with the development of chemo-resistance [16, 25]) by macrophages under the influence of tumor-derived factors [26]. The ability of recombinant IL-6 to induce protection against curcumin was verified on COLO-205 and ChaGo-K-1 (Fig. 5a). hCG was incapable of inducing the secretion of IL-6 or TNF-α from tumor cells (data not shown) or from PBAC (Fig. 5b). However, heightened levels of IL-6 and TNF-α were observed in supernatants of human PBACs incubated with hCG-primed tumor conditioned medium (Fig. 5b). Such PBAC supernatants, when incubated with naive COLO-205 and ChaGo-K-1 cells, induced resistance to curcumin-mediated loss of viability; anti-IL-6 neutralizing antibodies negated this protective effect (Fig. 5c). These results suggest chemo-resistance can arise as a consequence of the interaction between tumor cells and normal cells, with hCG being the intermediary.

### Effects of anti-hCG immunization and chemotherapy in tumor-bearing mice

Anti-hCG vaccination restricts the growth of murine tumors *in vivo* [27–29], data which validates the use of surrogate syngeneic immunization protocols.

**Fig. 6:**
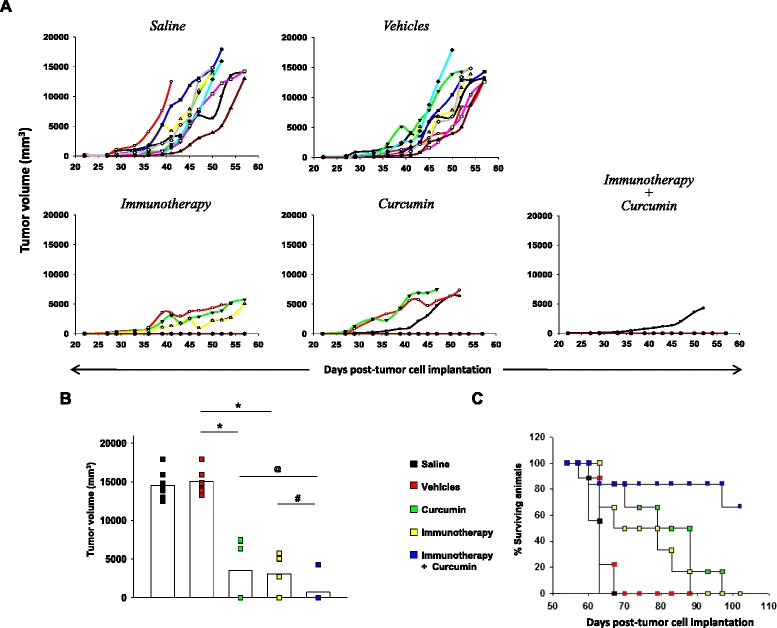
Enhanced anti-tumor effects of combination chemotherapy and anti-hCG immunotherapy. **a** Kinetics of tumor growth in individual C57BL/6 mice (n = 8) implanted with syngeneic LLC1 tumor cells. Data for control animals (treated with saline or vehicles) and for animals receiving anti-hCG immunotherapy, curcumin or immunotherapy + curcumin is shown. **b** Tumor volumes on Day 57 post-tumor cell implantation in C57BL/6 mice implanted with syngeneic LLC1 tumor cells treated with immunotherapy, curcumin or immunotherapy + curcumin. Each symbol represents an individual animal (*p < 0.001, ^@^p < 0.0001, ^#^p < 0.0002). **c** Kaplan-Meier analysis for C57BL/6 mice implanted with syngeneic LLC1 tumor cells receiving curcumin, immunotherapy or immunotherapy + curcumin. Control animals received either saline or vehicles. p < 0.0019, saline vs curcumin; p < 0.0228, saline vs immunotherapy; p < 0.0019, saline vs curcumin + immunotherapy; p < 0.0013, vehicles vs curcumin; p < 0.022, vehicles vs immunotherapy; p < 0.0013, vehicles vs immunotherapy + curcumin; p < 0.018, curcumin vs immunotherapy + curcumin; p < 0.007, immunotherapy vs immunotherapy + curcumin

All C57BL/6 mice injected with LLC1 cells generated tumors; administration of the vehicles had no effect on tumor incidence or volume. In animals receiving either immunotherapy (the hCGβ-TT vaccine) or chemotherapy (curcumin), tumor incidence was reduced to 50 %; tumors that did arise demonstrated significantly smaller volumes, as assessed on Day 57 post-tumor implantation. The consequences of combination immunotherapy and chemotherapy on tumor growth were also subsequently assessed. Co-administration had effects on multiple parameters; while tumor incidence was further reduced, mean tumor volumes were significantly lower than those observed upon individual immunotherapy or chemotherapy. Further, there was an extended lag period before the appearance of tumors (Fig. 6 a, b). Kaplan-Meier analysis re-iterated the benefits of the combinatorial approach on animal survival. Mortality rates in animals treated with the vehicles were similar to those observed in untreated animals, with all animals in both groups succumbing by Day 70. Curcumin treatment and anti-hCG vaccination significantly improved survival over these control groups when individually administered; there was no difference in animal survival between chemotherapy and immunotherapy. Importantly, treatment with immunotherapy in combination with chemotherapy led to significant improvements in survival over both treatments administered individually (Fig. 6c).

### Synergy between anti-hCG antibodies and curcumin

The simultaneous addition of anti-hCG antibodies and curcumin on ChaGo-K-1 cells induced significant inhibitory effects over the addition of individual moieties (Additional file 6: Figure S6A).

A graphical representation of the analysis for the determination of the Combination Index (C.I.) is depicted in Additional file 6: Figure S6B. IC_50_ values for curcumin and anti-hCG antibodies are plotted as (IC_50_ antibody, 0), (0, IC_50_ curcumin) and C_50_ antibody, C_50_ curcumin (for two representative data points) as dot plots. For the two data points, C.I. values were 0.699 (red) and 0.711 (green), values which constitute evidence of synergy, being less than one.

## Discussion

Growing evidence suggests that tumors secrete factors that can affect either tumor cells themselves or cells in the microenvironment to regulate growth and progression [17].

The demonstration of hCG in several non-trophoblastic tumors such as pancreatic and biliary cancer [4], urothelial cancer [5] and lung cancer [6] has spurred interest in patho-physiological roles the hormone might play, particularly since elevated levels of the hormone are often correlated with higher metastatic potential [11], unresponsiveness to drugs and poor patient prognosis [8, 9, 20]. Though the increase in the viability and proliferation of tumor cells upon exposure to exogenous hCG in this study are in line with similar findings by previous investigators, the molecular mechanisms linking the hormone to chemo-resistance (a major determinant of poor prognosis) have not been investigated in any detail, save for a study demonstrating the ability of hCG to induce resistance to cisplatin in ovarian cancer cells [30].

The current study provides substantial evidence of the chemo-protective effects of hCG. While tamoxifen has been widely employed in the treatment of human cancer, several studies have demonstrated the toxic effects curcumin on tumor cells of various lineages; it is an antioxidant and anti-inflammatory agent, and an inhibitor of NF-kappa B. Interestingly, curcumin has also been demonstrated to enhance sensitivity of tumor cells to a variety of drugs and hence has been considered a “chemosensitizing” agent [31]. hCG also reduced etoposide- and 5-FU- mediated loss of tumor cell viability (Additional file 7: Figure S7A). Two additional read-outs supported the idea that hCG has trophic effects on tumor cells. Firstly, hCG ameliorated the suppression of proliferation (as assessed by the uptake of [^3^H]-Thymidine) induced by curcumin, tamoxifen and 5-FU (Additional file 7: Figure S7B). Secondly, while 5-FU caused a decrease in basal levels of secretion of IL-8 (a cytokine associated with tumorigenesis) from COLO-205 cells, pre-incubation with hCG prevented such decreases (Additional file 7: Figure S7C). Since the drugs employed in this study act via different mechanisms to induce cytotoxicity, the study demonstrated that the protective effects of hCG were wide-ranging, generic and not restricted to a specific pathway.

While several genes have been implicated in chemoresistance, it was not the primary aim of this manuscript to assess the effects of hCG on them all. Rather, this study had a more focussed objective. The genes selected for study in this work were primarily chosen on the basis of two criteria: Firstly, all had previously been shown to be modulated by hCG (in reproductive tissues but not in cancer cells) and secondly, all had also been shown to play a role in chemoresistance (mediated by moieties other than hCG, either in reproductive tissues or in cancer cells). Application of these dual criteria resulted in the selection of genes that were of a more generic nature, not associated with a particular drug.

Incubation of tumor cells with hCG resulted in an increased expression of c-FLIP, an endogenous inhibitor of caspase 8. Since caspase 8 acts to up-regulate levels of caspase 3, these results are in line with the observed decrease in the conversion of pro-caspase 3 to caspase 3 upon incubation of tumor cells with hCG. Increase in BCL-2 upon hCG treatment in human colorectal and lung cancer cells in the current study suggests that inhibition of the mitochondrial pathway of apoptosis also possibly contributes to hCG-induced protection from drug-induced tumor cell death, although this postulate awaits formal verification.

hCG-induced effects on tumor cells appear to be the result of multiple collaborative pathways. Down-stream events can be postulated to occur post the proliferative responses induced by hCG, since uncontrolled proliferation can increase oxidative stress, endoplasmic reticulum (ER) stress and genomic instability-induced stress [32], a likely result of which is apoptosis. Tumor cells respond to such threats by up-modulation of ER stress regulators like BCL-2, HIF-1α, PARP-1, HSP27, HO-1 and PON2, all molecules shown to be up-modulated in tumor cells upon the addition of hCG in this study; hCG also enhanced levels of IAPs such as SURVIVIN, cIAP-1 and XIAP, known contributors to chemo-resistance. These results imply that hCG may act as an anti-stress regulator. The reversal of hCG-induced chemo-resistance in cells depleted of NRF-2 (a transcription factor upstream of HIF-1α and HO-1) and SURVIVIN in the current study suggests that hCG can exert its influence on both the proximal and distal ends of the apoptotic pathway. The fact that hCG provides protection against drugs which act via distinct mechanisms, as well against nutrient exhaustion-induced death (data not shown), supports this conclusion.

Increasing evidence links TLR signalling with tumorigenesis and the development of chemo-resistance [13, 22, 33]. In the current study, hCG increased the transcription and expression of TLR1, TLR3, TLR8 and TLR9; interestingly, and the combined addition of hCG with ligands for these TLRs (along with many others) resulted in enhanced chemo-protective effects, beyond those observed when hCG and the TLR ligands were individually employed. Because of the nature of some of the ligands employed in this study as well as the fact that some TLRs exist as signalling heterodimers, the results may not be always attributable to individual TLRs. For example, while HKLM appears to be an exclusive TLR2 agonist, Pam3CSK4 binds the TLR1/TLR2 dimer and FSL-1 the TLR2/TLR6 dimer; outcomes using Pam3CSK4 and FSL-1 may therefore be a consequence of TLR2 triggering. Regardless of these fine distinctions and in light of the data with other ligands, it does appear that NF-κB may comprise a central event, where both MyD88-dependent (all TLRs with the exception of TLR3) and TRIF-dependent (TLR3 and TLR4) pathways converge [34]. The global enumeration of genes that are modulated in response to dual ligand stimulation is underway.

Data obtained upon analysis of signalling events in cells exposed to hCG, TLR ligand and chemotherapeutic drug is strongly suggestive of tri-molecular synergy in some instances. Of particular interest in this regard were signalling synergies between hCG and the TLR9 ligand ODN2006 for JNK phosphorylation and between hCG and the TLR8 ligand ssRNA40 for ERK phosphorylation, given the fact that both TLR8 and TLR9 were upmodulated by hCG. Based on currently available and emerging data, the following scenario can be envisaged: Assault by chemotherapeutic drugs induces the release endogenous TLR ligands from cells as they die. In the cells that survive, hCG-mediated up-modulation of TLR expression contributes to enhanced hCG-TLR intracellular signalling; the consequence of these events is the creation of an environment characterized by heightened levels of stress regulatory mediators which drive chemo-resistance (Additional file 8: Figure S8). The sustained activation of MAP kinase signalling pathways, which may contribute to these events, may have further implications as well; tumorigenesis is often characterized by enhanced and sustained signalling via these pathways [35, 36]. Indeed, inhibition of the JNK and ERK signaling pathways are considered valid therapeutic strategies. Ligands capable of triggering such events in tumor cells (particularly those not arising as a result of mutational events) are of obvious interest.

Of the many accessory cells known to augment tumor progression, macrophages are of particular interest [37]. Previous studies have suggested a role for IL-6 in the development of chemo-resistance [16, 25]. Tumor-derived versican (a proteoglycan which sequesters chemokines and acts as a chemo-attractant for inflammatory cells in the tumor milieu) can stimulate resident macrophages to secrete IL-6 and TNF-α in a TLR-2 dependent manner [26]. Previous work in the lab has demonstrated the ability of hCG to enhance the secretion of versican from tumor cells, resulting in the release of these inflammatory cytokines in a TLR-2 dependent manner (data not shown). The current work demonstrates that the enhanced levels of macrophage-elicited IL-6 can mediate chemo-resistance. These results suggest that, in addition to mediating direct chemo-protective effects, hCG can also initiate cross-talk between tumor cells and macrophages to induce the generation of chemo-protective cytokines.

On-going studies have established that implantation of tumor cells in mice transgenic for βhCG results in increased tumor incidence and higher tumor volume. In addition, transcription of several of the genes identified in this study is up-modulated in tumors implanted in these animals (data not shown).

In view of the known and suspected roles of hCG in tumorigenesis, anti-hCG vaccination is increasingly considered an attractive option. A vaccine based on the C-terminal region of hCG induced beneficial effects in patients of colorectal cancer [38]. Previous studies have also described the ameliorating effects on the reproductive axis of anti-hCG immunization in hCGβ transgenic mice which express ovarian hyperplasia and pituitary tumors [39]. Most other work in the area involves the use of surrogate murine systems [27–29]. One of these studies [28] provided evidence for adjuvantic and anti-tumor effects of MIP in anti-hCG vaccination, work that was based on previous published reports on the anti-tumor potency of MIP in mice [40] and in humans [41]. While IFA has often been employed as an adjuvant in humans with encouraging results [42], the other components in the formulations employed in this study have been approved for use in humans, with an analogous vaccine having previously demonstrated the ability to break tolerance towards hCG and exert anti-fertility effects in women [43].

Concurrent chemotherapy is believed to potentiate immunotherapeutic responses, possibly by increasing inflammation in the target area [44]. Conversely, immunotherapy, particularly directed against a soluble, potential tumor-promoting moiety like hCG that induces chemo-resistance, can potentially enhance the therapeutic efficacy of chemotherapy as well. The current study, using a validated murine model, suggests that the combination of chemo-therapy and immunotherapy offers distinct advantages; add-on anti-hCG immunotherapy improved three critical parameters of tumorigenesis in animals treated with curcumin: tumor incidence, tumor volume and animal survival. Quite conceivably, such combination regimens would permit reduction in chemotherapeutic drug dose, thereby serving to also ameliorate associated toxicities.

## Conclusions

Tumor-derived gonadotropin has been associated with reduced survival. The novelty of the current study lies in the elucidation of some of the mechanistic details of the hormone’s tumor-protective effects in the face of drug assault. The data uniquely demonstrates that hCG elicits pro-survival signals which interfere with the apoptotic cascade and can enhance the ability to counter cellular stress. That the additional presence of TLR ligands was shown to greatly enhance chemoresistance supports the testable, hitherto unconsidered postulate that dying tumor cells (a potential source of endogenous TLR ligands) may engender increased resistance in tumor cells that survive. The collaborative elicitation of IL-6 from monocytes presents an alternative mechanism of hCG-mediated chemo-resistance. The data also suggest that combination immunotherapy-chemotherapy protocols, using moieties and materials already in human use, may result in substantial beneficial effects in patients carrying gonadotropin-sensitive tumors.

## Additional files

Additional file 1: Figure S1. Sequences of primers employed in RT-PCR analysis. (PDF 159 kb) Additional file 2: Figure S2. Increased transcription and expression of TLRs in COLO-205 tumor cells incubated with hCG. Semi-quantitative RT-PCR (left panels) and Western blot (right panels) analysis of TLRs upon incubation of COLO-205 cells with medium or hCG. β-ACTIN was employed as loading control. (PDF 24 kb) Additional file 3: Figure S3. hCG and TLR ligands enhance chemo-resistance. Cell viability analysis on COLO-205 cells incubated with hCG, individual TLR ligands and tamoxifen in combination. H: hCG; P: Pam3CSK4; HK: HKLM; PI: Poly I:C; L: LPS; FL: Flagellin; F: FSL-1; I: Imiquimod; ss: ssRNA40; O: ODN2006. Means ± SEM of three independent experiments (with three replicates) are shown. In each case, data has been individually normalized to control cultures. (^@^p < 0.0006 vs medium; ^#^p < 0.0001, ^$^p < 0.002 vs hCG + tamoxifen; ^&^p < 0.0001; ^%^p < 0.004, ^^^p < 0.02 vs hCG + TLR ligand). (PDF 24 kb) Additional file 4: Figure S4. hCG increases mRNA and expression levels of molecules associated with chemo-resistance, apoptosis and cell stress in tumor cells. A. Semi-quantitative RT-PCR of molecules associated with chemo-resistance upon incubation of COLO-205 cells with medium or medium supplemented with hCG. β-ACTIN was employed as loading control. B. Western blot of molecules associated with chemo-resistance upon incubation of COLO-205 cells with medium or medium supplemented with hCG. β-ACTIN was employed as loading control. C. Flow cytometric analysis of molecules associated with chemo-resistance upon incubation of COLO-205 cells with medium or medium supplemented with hCG. Cells were permeabilized and stained with the respective antibodies. Filled red profiles indicate secondary antibody controls, green profiles indicate control cells and blue profile cells incubated with hCG. Data representative of four independent experiments. (PDF 24 kb) Additional file 5: Figure S5. “Off target” effects of siRNAs (at 120 pmol) against SURVIVIN, HIF-1α, HO-1 and NRF-2, when individually employed, against the other three targets. β-ACTIN was employed as loading control. scRNA: scrambled RNA. (PDF 24 kb) Additional file 6: Figure S6. The effects of anti-hCG antibodies and curcumin, added individually and in combination, on the viability of ChaGo-K-1 cells. A. Representative MTT analysis at an antibody dose of 2 μg/ml and the indicated doses of curcumin. Means ± SEM of three independent experiments (with three replicates) are shown. In each case, data has been individually normalized to control cultures.*p < 0.006; **p < 0.0008; ***p < 0.0007; ^#^p < 0.04; ^##^p < 0.0003; ^###^p < 0.0001. Isotype control antibodies: Negative control for anti-hCG antibodies. B. Assessment of synergistic effects of curcumin and anti-hCG antibodies on the viability of ChaGo-K-1 cells. The Combination Index (C.I.) was calculated (as described in Materials and Methods) for several data points, two of which are depicted. Red data point: 0.699; Green data point: 0.711. (PDF 24 kb) Additional file 7: Figure S7. Effects of hCG on drug-induced loss of cell viability, proliferation and cytokine secretion. A. Effects on 5-Fluorouracil- (top panel) or Etoposide- (bottom panel) -induced decrease in the viability of COLO-205 cells. Means ± SEM of four independent experiments (with three replicates) are shown. In each case, and for both control cultures and cultures containing hCG, data has been individually normalized to cells plus vehicle control (VC). (^$^p < 0.0009, ^#^p < 0.005, *p < 0.0001, ^%^p < 0.0006 vs cells not exposed to hCG). B. Effects on drug-induced decreases in the incorporation of [^3^H]-Thymidine in LLC1 cells. (*p < 0.0001 vs cells not exposed to hCG) VC: Vehicle control. C. Effects on 5-FU -induced decreases in the secretion of IL-8 from COLO-205 cells. “Medium” refers to RPMI 1640 with 10 % fetal bovine serum. TCM: Tumor-conditioned medium. For A-C, means ± SEM of four independent experiments (with three replicates) are shown (^<^p < 0.0002 vs control cells; ^#^p < 0.0003 vs 5-FU treated cells). (PDF 24 kb) Additional file 8: Figure S8. Postulated tri-molecular synergy between chemotherapeutic drugs, TLR ligands and hCG in the development of chemo-resistance in tumor cells. Upward arrows indicate up-modulation. See text for details. (PDF 201 kb)

## Abbreviations

ERK: Extracellular signal-regulated kinase
hCG: Human Chorionic Gonadotropin
HO-1: Hemeoxygenase 1
IL-6: Interleukin 6
TLR: Toll-like receptor
TNF: Tumor necrosis factor
