# Supplementary material for: Gonadotropin-mediated chemoresistance: Delineation of molecular pathways and targets
Source: BMC Cancer. 2015 Nov 25;15:931. doi: 10.1186/s12885-015-1938-x (PMC4660813; doi:10.1186/s12885-015-1938-x)
Supplement: Additional file 1: Figure S1. — Sequences of primers employed in RT-PCR analysis. (PDF 159 kb) [file 12885_2015_1938_MOESM1_ESM.pdf]

Gene	Forward primer	Reverse primer
HIF-1A	CCCCAGATTCAGGATCAGACA	CCATCATGTTCCATTTTTCGC
SURVIVIN	CCACCGCATCTCTACATTCA	TATGTTCCTCTATGGGGTCG
C-FLIP	GAGGTTGAGGGACTTGGCATG	TCAGCAGGACCCTATAATCAG
PARP-1	CTACTCGGTCCAAGATCGCC	TTGAAAAAGCCCTAAAGGCTCA
BCL-2	CGACTTCGCCGAGATGTCCAGCCAG	ACTTGTGGCCCAGATAGGCACCCAG
KLK-10	GATCACCTGCTGCTTCTTC	CACTCTGGCAAGGGTCCTG
NRF-2	GCCAGCTGAACTCCTTAGAC	GATTCGTGCACAGCAGCA
HO-1	AACCTCCAAAAGCCCTGAGT	CACCCCAACCCTGCTATAAA
TLR-1	CGTAAAACTGGAAGCTTTGCAAGA	GGACTTATTTGGAATGGCCCAAGG
TLR-2	GTGGCCAGCAGGTTCAGGATG	AGGACTTTATCGCAGCTCTCAG
TLR-3	ATTGGGTCTGGGAACATTTCTCTTC	GCGAAGAGGAATGTTTAAATCTCAC
TLR-4	ACAGAAGCTGGTGGCTGTG	TCTTTAAATGCACCTGGTTGG
TLR-5	CATTGTATGCACTGTCACTC	CCACCACCATGATGAGAGCA
TLR-6	TAGGTCTCATGACGAAGGAT	CGGAGTTATTTGCAGTGGCC
TLR-7	AGTGTCTAAAGAACCTGG	CTTGGCCTTACAGAAATG
TLR-8	CAGAATAGCAGGCGTAACACATCA	CCCTTTGAATGCACCTGTGACATT
TLR-9	TTATGGACTTCCTGCTGGAGGTGC	TGGTCTTCGACAAAACGCAG
B-ACTIN	AAGAGAGGCATCCTCACCCT	ATCTCTTGCTCGAAGTCCAG
