# Supplementary figures and images for: Gonadotropin-mediated chemoresistance: Delineation of molecular pathways and targets
Source: BMC Cancer. 2015 Nov 25;15:931. doi: 10.1186/s12885-015-1938-x (PMC4660813; doi:10.1186/s12885-015-1938-x)

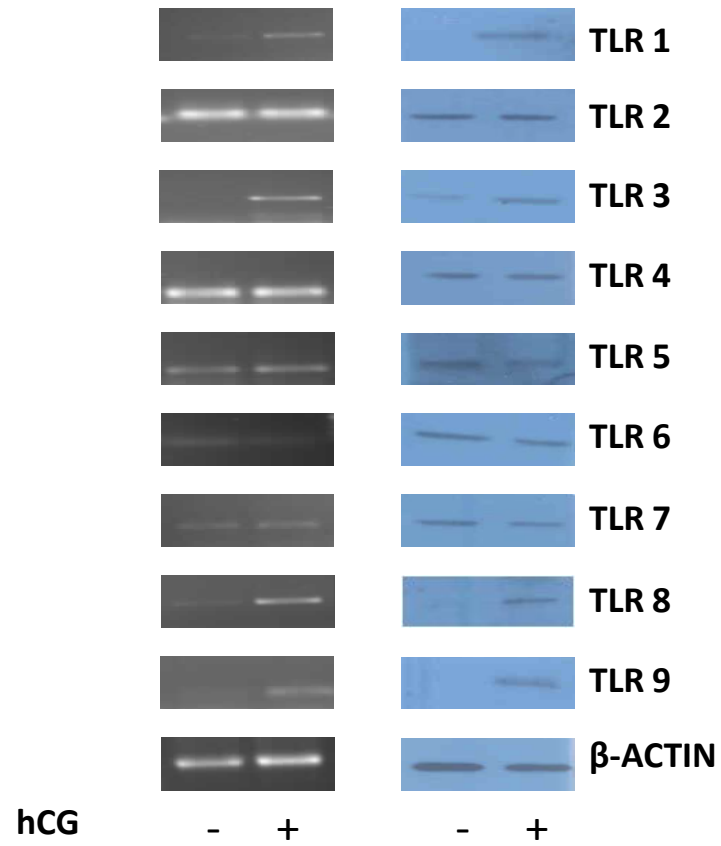

Supplement: Additional file 2: Figure S2. — Increased transcription and expression of TLRs in COLO-205 tumor cells incubated with hCG. Semi-quantitative RT-PCR (left panels) and Western blot (right panels) analysis of TLRs upon incubation of COLO-205 cells with medium or hCG. β-ACTIN was employed as loading control. (PDF 24 kb) [file 12885_2015_1938_MOESM2_ESM.pdf]

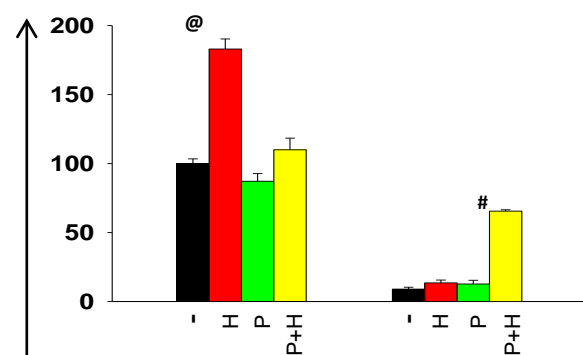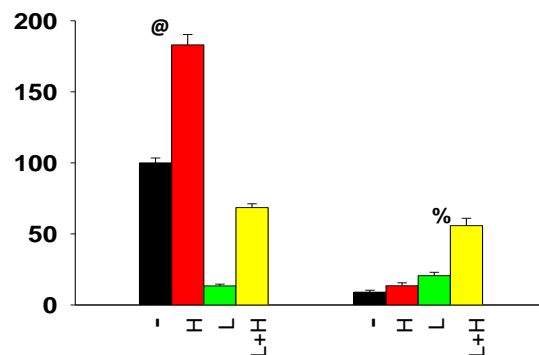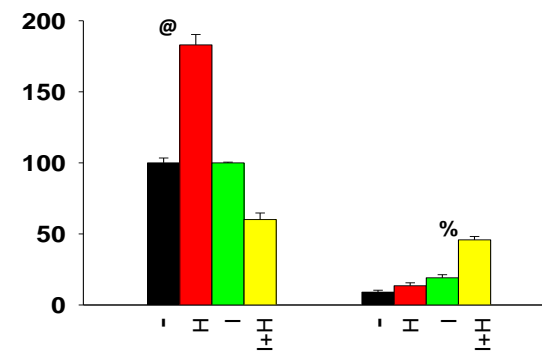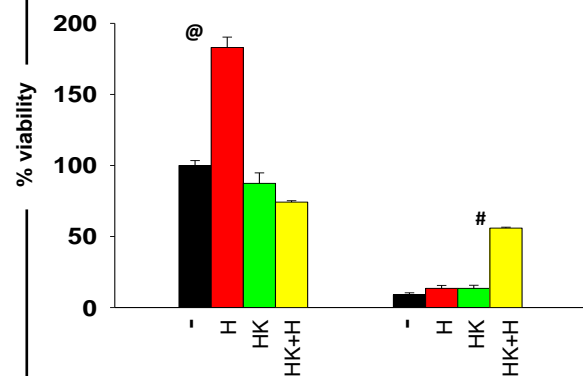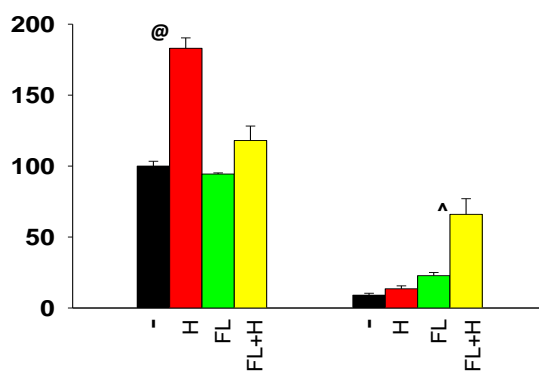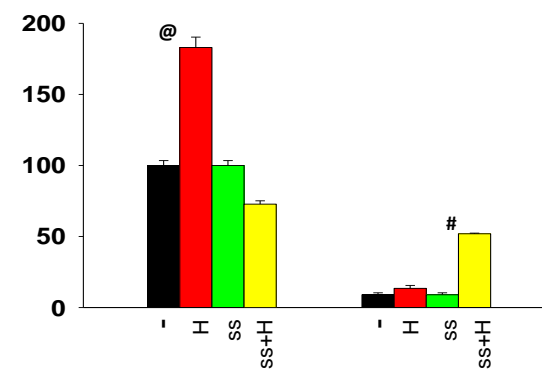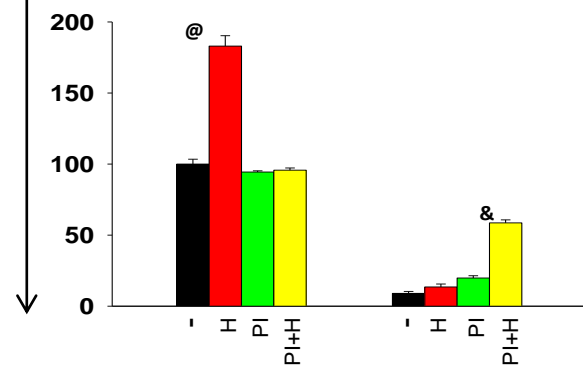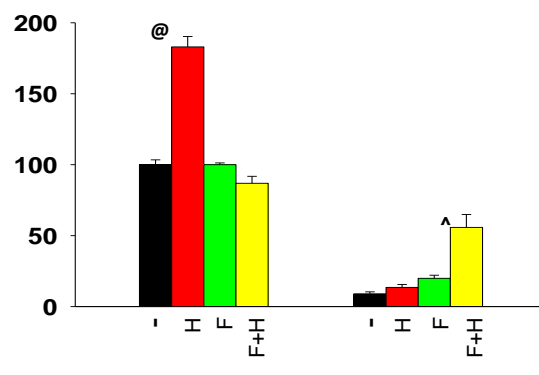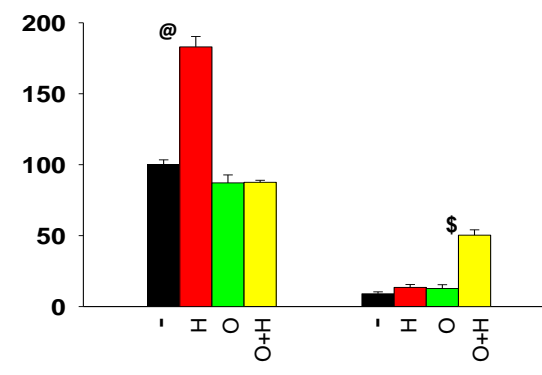

-

Tamoxifen

-

Tamoxifen

-

Tamoxifen

Supplement: Additional file 3: Figure S3. — hCG and TLR ligands enhance chemo-resistance. Cell viability analysis on COLO-205 cells incubated with hCG, individual TLR ligands and tamoxifen in combination. H: hCG; P: Pam3CSK4; HK: HKLM; PI: Poly I:C; L: LPS; FL: Flagellin; F: FSL-1; I: Imiquimod; ss: ssRNA40; O: ODN2006. Means ± SEM of three independent experiments (with three replicates) are shown. In each case, data has been individually normalized to control cultures. (@p < 0.0006 vs medium; #p < 0.0001, $p < 0.002 vs hCG + tamoxifen; &p < 0.0001; %p < 0.004, ^p < 0.02 vs hCG + TLR ligand). (PDF 24 kb) [file 12885_2015_1938_MOESM3_ESM.pdf]

**A**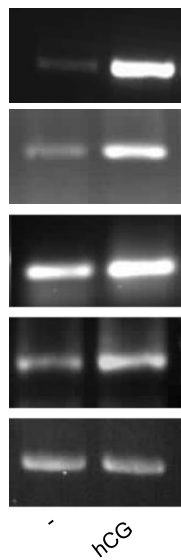**B**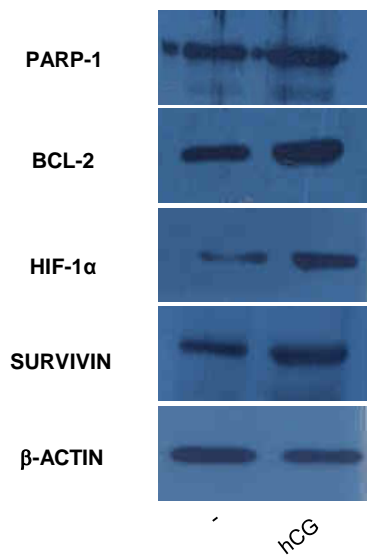**C**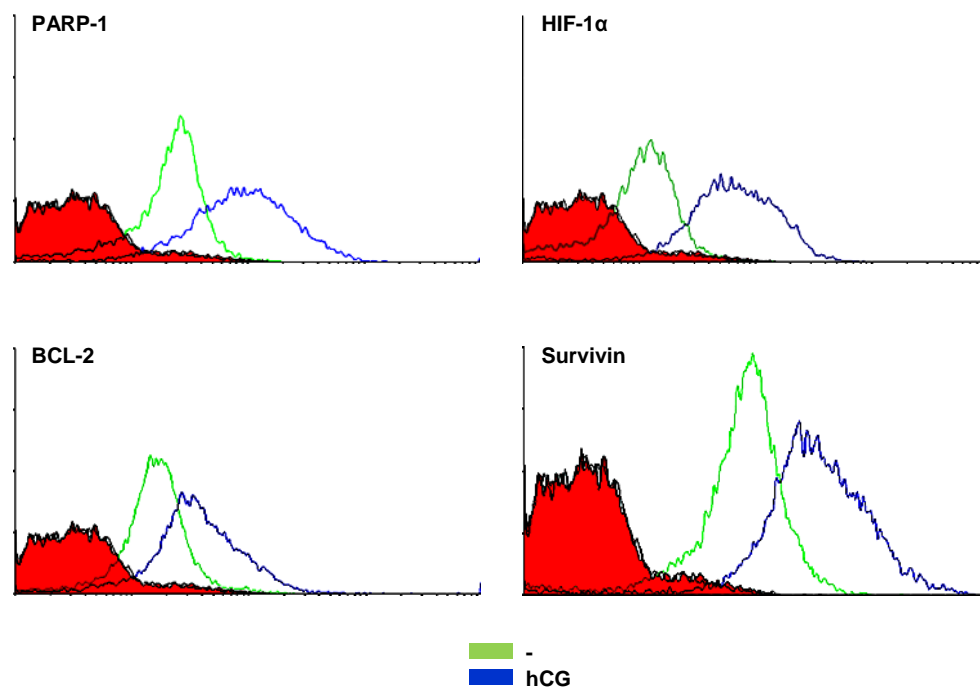

Supplement: Additional file 4: Figure S4. — hCG increases mRNA and expression levels of molecules associated with chemo-resistance, apoptosis and cell stress in tumor cells. A. Semi-quantitative RT-PCR of molecules associated with chemo-resistance upon incubation of COLO-205 cells with medium or medium supplemented with hCG. β-ACTIN was employed as loading control. B. Western blot of molecules associated with chemo-resistance upon incubation of COLO-205 cells with medium or medium supplemented with hCG. β-ACTIN was employed as loading control. C. Flow cytometric analysis of molecules associated with chemo-resistance upon incubation of COLO-205 cells with medium or medium supplemented with hCG. Cells were permeabilized and stained with the respective antibodies. Filled red profiles indicate secondary antibody controls, green profiles indicate control cells and blue profile cells incubated with hCG. Data representative of four independent experiments. (PDF 24 kb) [file 12885_2015_1938_MOESM4_ESM.pdf]

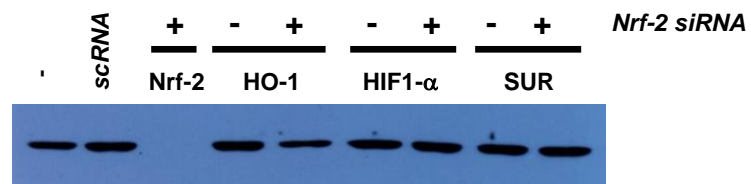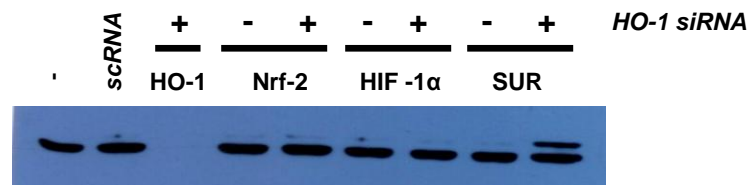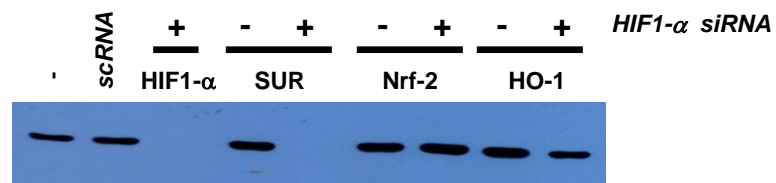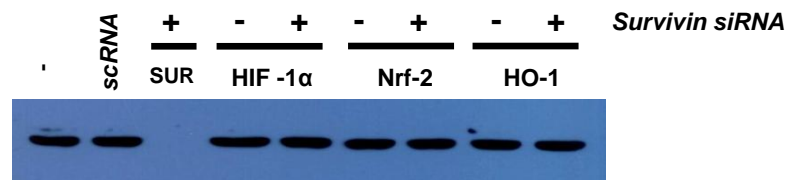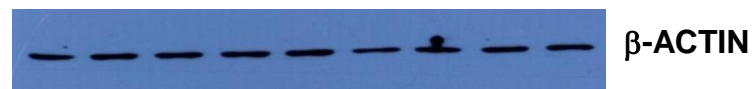

Supplement: Additional file 5: Figure S5. — “Off target” effects of siRNAs (at 120 pmol) against SURVIVIN, HIF-1α, HO-1 and NRF-2, when individually employed, against the other three targets. β-ACTIN was employed as loading control. scRNA: scrambled RNA. (PDF 24 kb) [file 12885_2015_1938_MOESM5_ESM.pdf]

**A**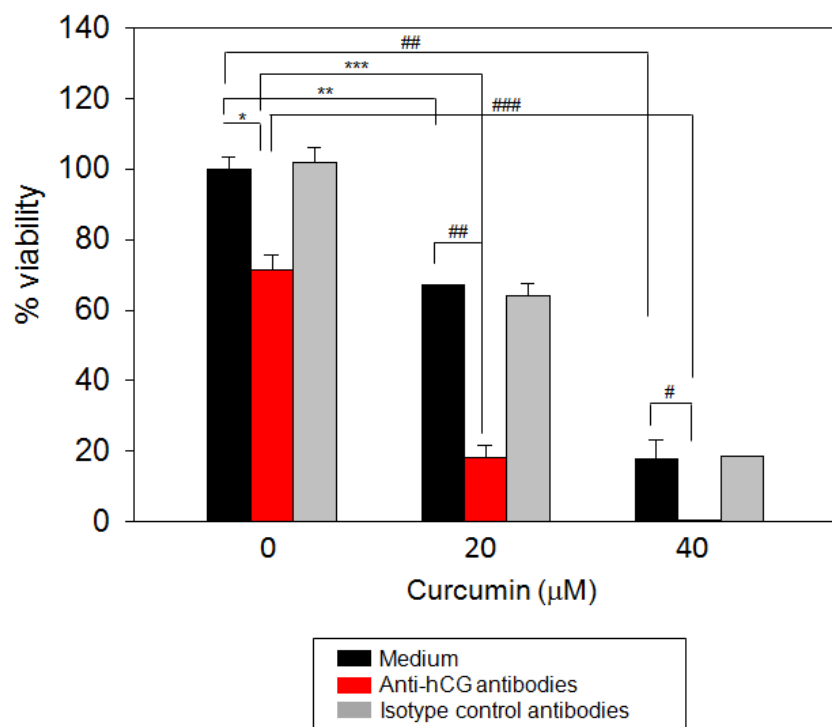**B**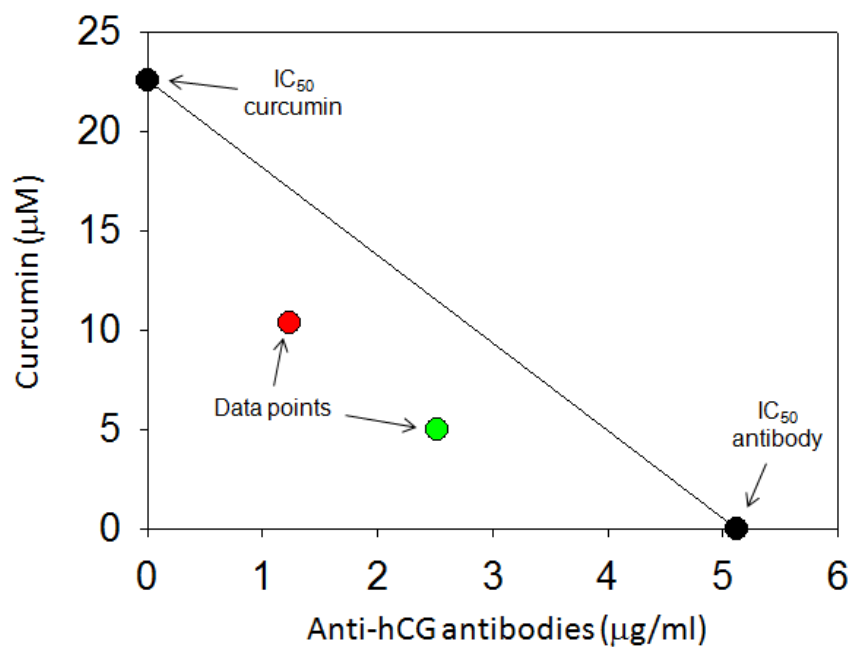

Supplement: Additional file 6: Figure S6. — The effects of anti-hCG antibodies and curcumin, added individually and in combination, on the viability of ChaGo-K-1 cells. A. Representative MTT analysis at an antibody dose of 2 μg/ml and the indicated doses of curcumin. Means ± SEM of three independent experiments (with three replicates) are shown. In each case, data has been individually normalized to control cultures.*p < 0.006; **p < 0.0008; ***p < 0.0007; #p < 0.04; ##p < 0.0003; ###p < 0.0001. Isotype control antibodies: Negative control for anti-hCG antibodies. B. Assessment of synergistic effects of curcumin and anti-hCG antibodies on the viability of ChaGo-K-1 cells. The Combination Index (C.I.) was calculated (as described in Materials and Methods) for several data points, two of which are depicted. Red data point: 0.699; Green data point: 0.711. (PDF 24 kb) [file 12885_2015_1938_MOESM6_ESM.pdf]

**A**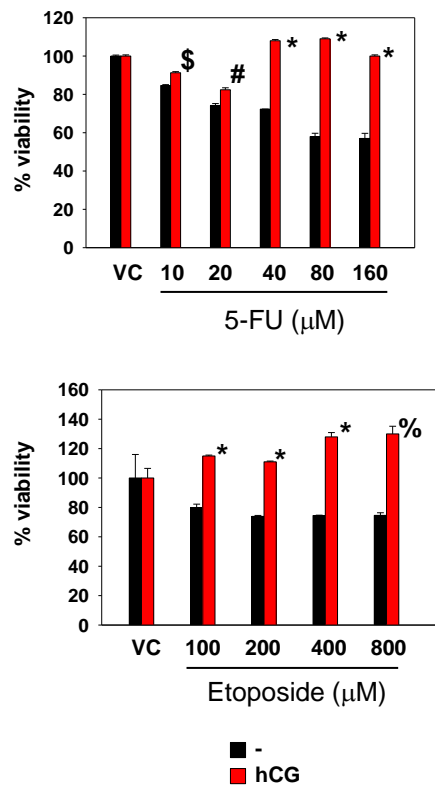**B**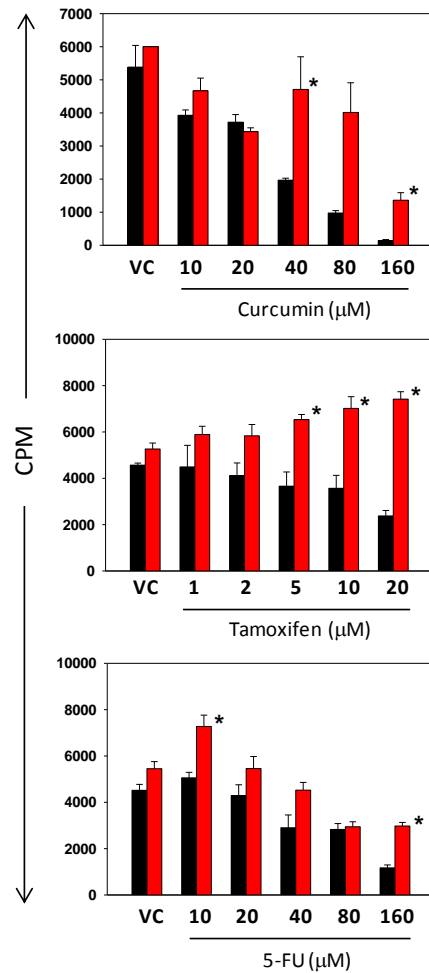**C**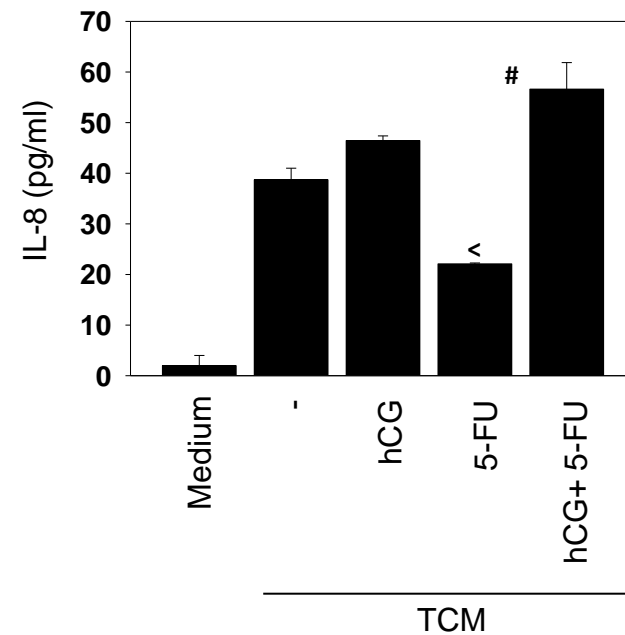

Supplement: Additional file 7: Figure S7. — Effects of hCG on drug-induced loss of cell viability, proliferation and cytokine secretion. A. Effects on 5-Fluorouracil- (top panel) or Etoposide- (bottom panel) -induced decrease in the viability of COLO-205 cells. Means ± SEM of four independent experiments (with three replicates) are shown. In each case, and for both control cultures and cultures containing hCG, data has been individually normalized to cells plus vehicle control (VC). ($p < 0.0009, #p < 0.005, *p < 0.0001, %p < 0.0006 vs cells not exposed to hCG). B. Effects on drug-induced decreases in the incorporation of [3H]-Thymidine in LLC1 cells. (*p < 0.0001 vs cells not exposed to hCG) VC: Vehicle control. C. Effects on 5-FU -induced decreases in the secretion of IL-8 from COLO-205 cells. “Medium” refers to RPMI 1640 with 10 % fetal bovine serum. TCM: Tumor-conditioned medium. For A-C, means ± SEM of four independent experiments (with three replicates) are shown (<p < 0.0002 vs control cells; #p < 0.0003 vs 5-FU treated cells). (PDF 24 kb) [file 12885_2015_1938_MOESM7_ESM.pdf]

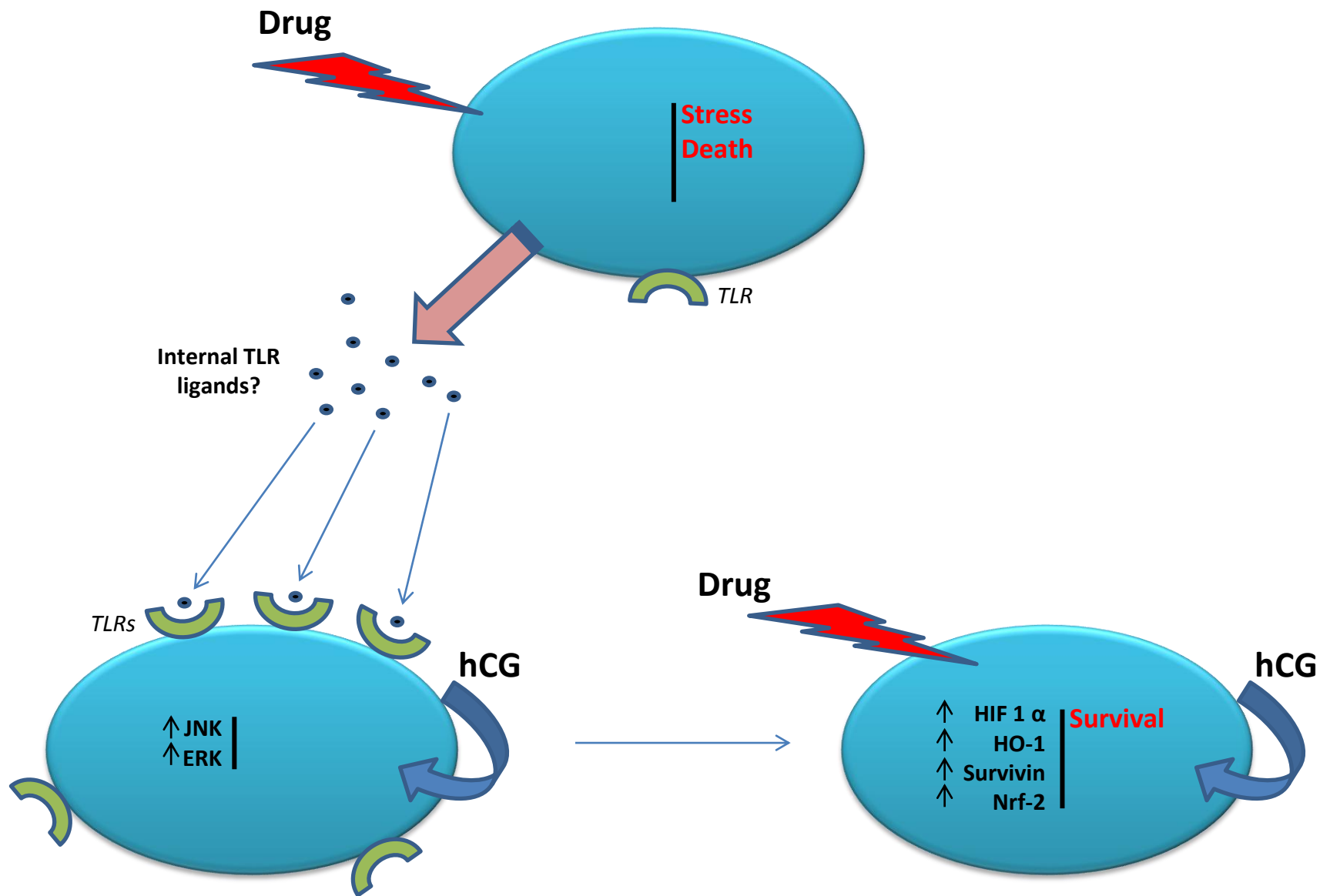

Supplement: Additional file 8: Figure S8. — Postulated tri-molecular synergy between chemotherapeutic drugs, TLR ligands and hCG in the development of chemo-resistance in tumor cells. Upward arrows indicate up-modulation. See text for details. (PDF 201 kb) [file 12885_2015_1938_MOESM8_ESM.pdf]
